# Editorial Comment: Validity of a patient-specific percutaneous nephrolithotomy (PCNL) simulated surgical rehearsal platform: impact on patient and surgical outcomes

**DOI:** 10.1590/S1677-5538.IBJU.2022.04.03

**Published:** 2022-07-19

**Authors:** Luciano A. Favorito, Natasha T. Logsdon

**Affiliations:** 1 Unidade de Pesquisa Urogenital Universidade do Estado do Rio de Janeiro Rio de Janeiro RJ Brasil Unidade de Pesquisa Urogenital - Universidade do Estado do Rio de Janeiro - Uerj, Rio de Janeiro, RJ, Brasil

Ahmed Ghazi ^1, 2^, Rachel Melnyk 3,Shamroz Farooq ^4^, Adrian Bell 3, Tyler Holler 3, Patrick Saba ^3^, Jean Joseph ^5^

1Simulation Innovation Lab, University of Rochester Medical Center, 601 Elmwood Avenue, Rochester, NY, 14642, USA;^2^Department of Urology, Simulation Innovation Lab, University of Rochester Medical Center, 601 Elmwood Avenue, Rochester, NY, 14642, USA;^3^ Simulation Innovation Lab, University of Rochester Medical Center, 601 Elmwood Avenue, Rochester, NY, 14642, USA;^4^University of Rochester School of Medicine and Dentistry, 601 Elmwood Avenue, Rochester, NY, 14642, USA;^5^Department of Urology, Simulation Innovation Lab, University of Rochester Medical Center, 601 Elmwood Avenue, Rochester, NY, 14642, USA

World J Urol. 2022 Mar;40(3):627-637.

DOI: 10.1007/s00345-021-03766-7 | ACCESS: 34165633

## COMMENT

In the past the endocast model confection was the most important method to study the intra-renal anatomy in humans and in anatomic models ( 1 - 4 ). Technological Advances in last year’s provide a great advance in the development of simulators for surgical training and recently in the Int Braz J Urol some papers studied this kind of translational anatomical studies ( 5 ).

Percutaneous nephrolithotomy training using simulation is very important to the young urologists and to all surgeons who can have multiple attempts and opportunity for trial-and-error learning. In the present paper the authors evaluate the impact of preoperative high-fidelity patient-specific percutaneous nephrolithotomy hydrogel simulations on surgical and patient outcomes using amazing figures.

This paper shows the importance of the translational research and anatomy for urological practice and for the training of urologists. The authors conclude that patient-specific procedural rehearsal is effective reducing the experience curve for a complex endourological procedure, resulting in improved surgical performance and patient outcomes.

The paper concludes that penile allotransplantation represents a revolutionary technique in the management of penile loss. The inclusion of external pudendal artery anastomoses appears to have prevented any form of penile skin necrosis and anastomosis of the corpora cavernosa appears sufficient for restoration of erectile function independent of the cavernous artery.
